# Druggability Evaluation of the Neuron Derived Orphan Receptor (NOR‐1) Reveals Inverse NOR‐1 Agonists

**DOI:** 10.1002/cmdc.202200259

**Published:** 2022-07-07

**Authors:** Daniel Zaienne, Silvia Arifi, Julian A. Marschner, Jan Heering, Daniel Merk

**Affiliations:** ^1^ Department of Pharmacy Ludwig-Maximilians-Universität München Butenandtstr. 5–13 81377 Munich Germany; ^2^ Institute of Pharmaceutical Chemistry Goethe University Frankfurt Max-von-Laue-Str. 9 60438 Frankfurt Germany; ^3^ Fraunhofer Institute for Translational Medicine and Pharmacology ITMP Theodor-Stern-Kai 7 60596 Frankfurt Germany

**Keywords:** NR4A3, transcription factor, nuclear receptor, neurodegeneration, fragment screening

## Abstract

The neuron derived orphan receptor (NOR‐1, NR4A3) is among the least studied nuclear receptors. Its physiological role and therapeutic potential remain widely elusive which is in part due to the lack of chemical tools that can directly modulate NOR‐1 activity. To probe the possibility of pharmacological NOR‐1 modulation, we have tested a drug fragment library for NOR‐1 activation and repression. Despite low hit‐rate (<1 %), we have obtained three NOR‐1 ligand chemotypes one of which could be rapidly expanded to an analogue comprising low micromolar inverse NOR‐1 agonist potency and altering NOR‐1 regulated gene expression in a cellular setting. It confirms druggability of the transcription factor and may serve as an early tool to assess the role and potential of NOR‐1.

## Graphical abstract

**Drug fragment screening** and subsequent SAR evaluation identified an inverse agonist of the neuron derived orphan receptor (NOR‐1) for which chemical tools are rare. The inverse agonist blocked the interaction of NOR‐1 with the co‐regulator SMRT and enhanced expression of the c‐MYC proto‐oncogene (MYC) which is directly suppressed by NOR‐1 thus confirming inverse NOR‐1 agonism in cellular setting.

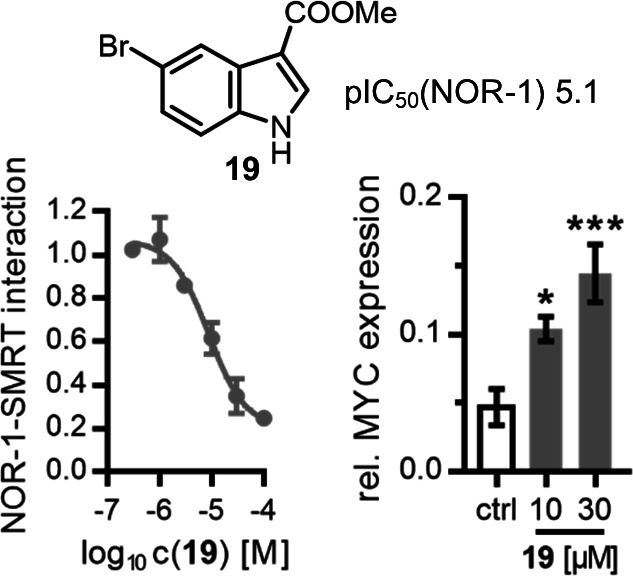

## Introduction

The transcription factor neuron derived orphan receptor (NOR‐1, NR4A3) is one of the least studied nuclear receptors. Its role and function are elusive and NOR‐1 ligands are lacking.^[1]^ The receptor is present in neurons throughout the spinal cord and the brain and mainly found in the hippocampus, cerebellum, cerebral neocortex, amygdala and dopaminoceptive areas like nucleus accumbens, striatum, olfactory tubercle, cingular and prefrontal cortices.[^2^, ^3^] Additionally, NOR‐1 is expressed in the heart and skeletal muscles and at low levels in vascular tissues and resting vascular cells.[^4^, ^5^] Apart from its inability to dimerize with RXR, NOR‐1 shares many characteristics with Nur77 (NR4A1) and Nurr1 (NR4A2), the other two human members of the nerve growth factor‐induced clone B (NGFI−B) nuclear receptor subfamily.^[6]^ Since NOR‐1 exhibits constitutive activity and lacks an accessible pocket in the canonical ligand binding region of nuclear receptors, its transcriptional activity mainly depends on its expression level.^[4]^ The possibility of modulating NOR‐1 activity with small drug‐like molecules ‐ its druggability ‐ remains elusive.

The limited knowledge on NOR‐1 suggests a potential involvement of NOR‐1 in neurodegenerative diseases.[^1^, ^2^, ^7^, ^8^] The transcription factor appears to play a critical role during CNS development and knockout studies provide preliminary evidence that NOR‐1 is also important for neuronal cell survival.^[1]^ Accumulation of NOR‐1 in Lewy bodies in Parkinson's Disease (PD) patients and in neuronal cytoplasmic inclusions in multiple system atrophy (MSA) has been detected, and the receptor has been shown to act as mediator of cyclic AMP response element‐binding protein (CREB)‐induced neuroprotection confirming an involvement of NOR‐1 in neuroprotection and neurodegeneration.[^2^, ^7^, ^8^] Moreover, several lines of evidence also point to an important role of NOR‐1 in cancer,[^9^, ^10^] as well as vascular biology, immunity, inflammation and lipid and glucose homeostasis.^[4]^

Only few molecules modulating the activity of NOR‐1 have been discovered so far.[^11^, ^12^] The anti‐inflammatory and antineoplastic drug 6‐mercaptopurine (6‐MP) enhanced NOR‐1 activity but was found to require the N‐terminal activation function 1 (AF‐1) of NOR‐1 for activity and does not bind to the ligand binding domain (LBD).^[12]^ The eicosanoid prostaglandin A2 (PGA2), in contrast, weakly activates NOR‐1 through direct interaction with its LBD.^[11]^ Both compounds are insufficient to study the pharmacological potential of the orphan nuclear receptor NOR‐1, however, and more potent and selective chemical tools are needed to investigate NOR‐1 as a potential therapeutic target. To further assess the druggability of NOR‐1 and explore the chemical space of its ligands, we have employed a chemically diverse fragment library to screen for potential NOR‐1 modulators. Three scaffolds (hit‐rate <1 %) were confirmed as NOR‐1 ligands including two inverse agonists and a weak agonist. Preliminary structure‐activity relationship (SAR) elucidation suggests potential of these compounds as leads for NOR‐1 modulator development and resulted in the discovery of an inverse NOR‐1 agonist with low micromolar potency. Its in vitro characterization confirmed cellular target engagement and revealed NOR‐1 co‐regulator interactions potentially involved in NOR‐1 modulation. Our results thus demonstrate the possibility of pharmacological NOR‐1 modulation and provide leads for NOR‐1 ligand development as well as an early chemical tool for further studies.

## Results and Discussion

**Figure 1:**
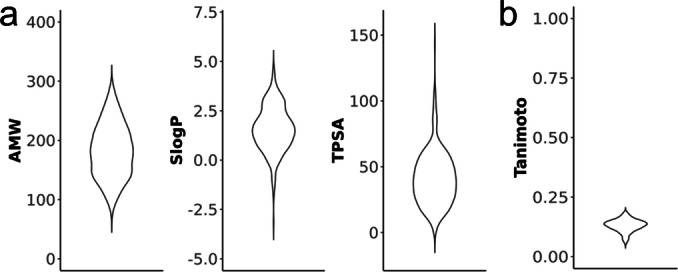
The screening library comprised fragment structures with low molecular weight as well as favorable SlogP and TPSA distribution (a), and provided high chemical diversity as illustrated by low average pairwise Tanimoto similarity computed on Morgan fingerprints (b).

To rapidly probe druggability of NOR‐1 and identify potential ligand scaffolds, we have established a robust reporter gene assay to monitor NOR‐1 activity and screened a medium size fragment library for NOR‐1 modulation. The screening assay was based on the Gal4 hybrid system^[13]^ and employed a chimeric receptor composed of the human NOR‐1 LBD and the Gal4 DNA binding domain from yeast. Firefly luciferase under the control of five tandem repeats of the Gal4 response element served as reporter gene and constitutively expressed renilla luciferase was used for normalization and toxicity monitoring. Control experiments were conducted with the ligand‐independent transcriptional inducer Gal4‐VP16[^14^, ^15^] (replacing Gal4‐NOR‐1) to exclude non‐specific effects. The commercially available fragment screening library (core set of the Prestwick drug fragments library) comprised 480 compounds with favorable fragment properties (Figure 1a) and offered high structural diversity as illustrated by low average pairwise Jaccard‐Tanimoto similarity computed on Morgan fingerprints^[16]^ (median 0.13; Figure 1b) and by a total of 77 unique Murcko scaffolds.^[17]^ Importantly, this library has already yielded valuable ligands for other nuclear receptors including TLX,[^18^, ^19^, ^20^] Nurr1^[21]^ and HNF4α.^[22]^

**Figure 2:**
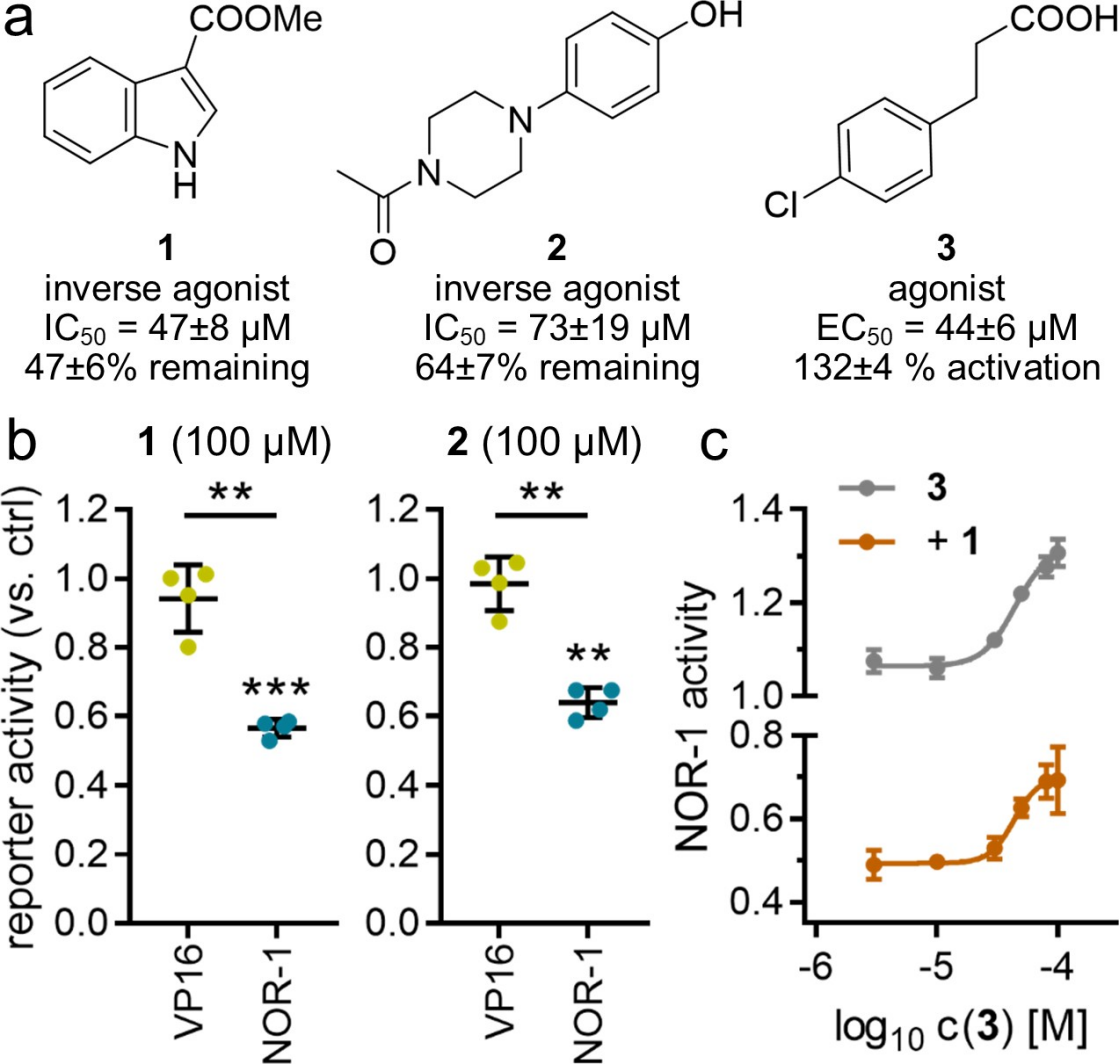
NOR‐1 modulators **1**–**3** discovered in the fragment screening. (a) Chemical structures of **1**–**3** and activity on NOR‐1 (EC_50_ and IC_50_ values as well as max. activation and remaining activity are the mean±SEM; n=4). (b) **1** and **2** caused no suppression of Gal4‐VP16 induced reporter expression but counteracted Gal4‐NOR‐1 supporting direct inverse NOR‐1 agonism. Data are the mean±SD relative reporter activity vs. 0.1 % DMSO in the respective setting; n=4. **p<0.01, ***p<0.001 (t‐test vs. 0.1 % DMSO treated cells or as indicated). (c) Competition experiments indicated different binding sites of the agonist **3** and the inverse agonist **1** since addition of a fixed concentration of **1** (100 μM) did not alter the EC_50_ value of **3**. Data are the mean±SD; n=3.

In the initial screening, the library was tested for NOR‐1 modulation in the cellular assay at 100 μM in two biologically independent repeats. 16 primary hits enhanced or decreased reporter activity indicative of potential NOR‐1 modulation and exhibited no toxic effect as observed by unaltered renilla luciferase activity. In control experiments involving the strong ligand‐independent transcriptional activator Gal4‐VP16^[14]^ only three of the primary hits (**1**‐**3**) showed no effect on Gal4‐VP16 induced reporter activity at 100 μM, however, and were thus fully profiled on NOR‐1. **1** and **2** acted as inverse agonists blocking constitutive NOR‐1 activity, and **3** weakly activated NOR‐1 (Figure 2a & b). Competition experiments suggested different binding sites (Figure 2c) since the addition of the inverse agonist **1** did not affect the EC_50_ value of **3** but shifted the curve downwards to lower NOR‐1 activities pointing to non‐competitive behavior and thus independent binding of the agonist **3** and the inverse agonist **1**.

Compared to previous screening campaigns of the same chemically diverse fragment library on nuclear receptors[^20^, ^21^, ^22^] and enzymes,^[23]^ the hit‐rate on NOR‐1 was low. This aligns with the fact that almost no NOR‐1 modulators have been reported to date and suggests that NOR‐1 is particularly restrictive in terms of ligand binding. Despite the low hit‐rate, our fragment screening approach has yielded three NOR‐1 modulator scaffolds demonstrating that NOR‐1 is druggable and can be controlled with small molecule ligands. The fragment hits **1** (SlogP 1.95), **2** (SlogP 1.06) and **3** (SlogP 2.36) have attractive physicochemical properties and are chemically diverse (0.136–0.152 pairwise Tanimoto similarity computed on Morgan fingerprints). These scaffolds may hence be valuable lead structures for NOR‐1 modulator development.

NOR‐1 acts as a constitutively active transcription factor and inverse agonists would thus be particularly useful as tool to study the receptor‘s roles in health and disease. Hence, we probed the potential of **2** (Table 1) and **1** (Table 2) as lead structures for inverse NOR‐1 agonist development and preliminarily studied their structure‐activity relationship (SAR) as NOR‐1 modulators.

**Table 1:** Activity of **2** and analogues on Gal4‐NOR‐1. Data are the mean±SD, n=4.

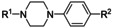			
ID	R^1^	R^2^	activity on NOR‐1
**2**	acetyl	−OH	inverse agonist IC_50_=73±19 μM (64±7 %)
**4**	−H	−OH	inactive (100 μM)
**5**	acetyl	−H	93±1 % NOR‐1 activity (100 μM)
**6**	−H	−Cl	120±6 % NOR‐1 activity (100 μM)
**7**	−H	−NO_2_	118±3 % NOR‐1 activity (100 μM)
**8**	−H	−NH_2_	124±2 % NOR‐1 activity (100 μM)
**9**	−H	−OCH_3_	inactive (100 μM)
**10**	−CH_3_	−NH_2_	inactive (100 μM)

**Table 2:** Activity of **1** and analogues on Gal4‐NOR‐1. Data are the mean±SD, n=4.

ID	structure	IC_50_ (NOR.1) (remaining activity)
**1**	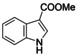	47±8 μM (47±6 %)
**12**	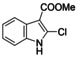	>100 μM
**13**	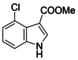	>100 μM
**14**	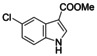	14±2 μM (12±4 %)
**15**	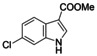	>100 μM
**16**	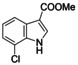	>100 μM
**17**	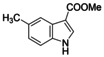	35±8 μM (2±7 %)
**18**	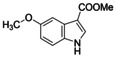	43±5 μM (13±4 %)
**19**	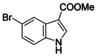	8±1 μM (7±3 %)
**20**	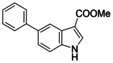	4±2 μM (49±5 %)

Removal of the acetyl moiety (**4**) or the phenolic hydroxyl group (**5**) from **2** caused a marked loss in inverse agonist activity. Interestingly, 4‐chlorophenylpiperazine (**6**), 4‐nitrophenylpiperazine (**7**) and 4‐aminophenylpiperazine (**8**) failed to block NOR‐1 activity but acted as weak agonists. 4‐Methoxyphenylpiperazine (**9**) was inactive and methylation of the weak agonist **8** in **10** resulted in a loss of activity, too. This preliminary SAR evaluation indicated that the N‐acetyl moiety and the phenolic hydroxyl group were both critical for inverse agonism of **2**. Moreover, weak NOR‐1 activation by several analogues suggested a possibility to also obtain agonists from the scaffold of **2**. Systematic extension of **2** may hence enable the development of potent NOR‐1 agonists and inverse agonists as chemical tools.

**Figure 3:**
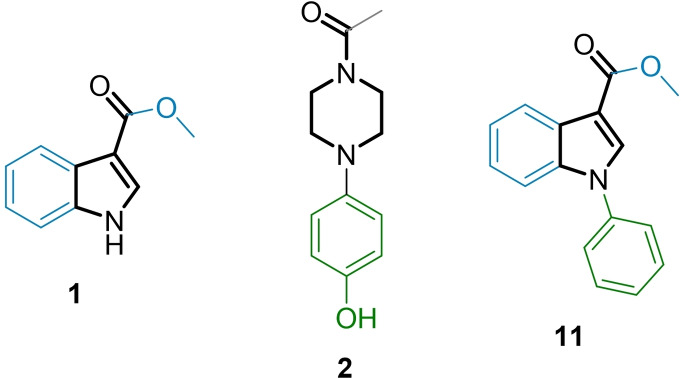
Structural similarity of inverse NOR‐1 agonists **1** and **2**, and the fused analogue **11**.

The inverse NOR‐1 agonists **2** and **1** shared structural features that suggested potential for fusion (Figure 3) prompting us to study the activity of the fused analogue **11** as NOR‐1 modulator. The combined design **11** was inactive on NOR‐1 at 100 μM, however, indicating that this strategy was not productive. Thus, we followed a systematic approach to obtain also preliminary insights in the SAR of the scaffold of **1** as inverse NOR‐1 agonist. To scan the binding site for space to accommodate structural extension, we first studied the effect of an additional chloro substituent in each free position of the small fragment skeleton (Table 2). A chlorine atom in 2‐ (**12**), 4‐ (**13**), 6‐ (**15**) or 7‐position (**16**) diminished activity on NOR‐1 suggesting little potential for major structural modifications in these positions. 5‐Chloro substitution (**14**), in contrast, was favored and led to a 3.5‐fold improvement in inverse NOR‐1 agonist potency highlighting extension in this region as potential avenue to structural optimization. The 5‐methyl (**17**) and 5‐methoxy (**18**) analogues were less active than **14** but still superior to the lead **1** especially in terms of inverse agonist efficacy therefore confirming substitution in 5‐position as favored. Introduction of bulkier bromo (**19**) and phenyl (**20**) substituents in 5‐position boosted potency to single‐digit micromolar IC_50_ values. Methyl 5‐bromoindole‐3‐carboxylate (**19**) comprised superior efficacy reducing NOR‐1 activity to <10 %.

**Figure 4:**
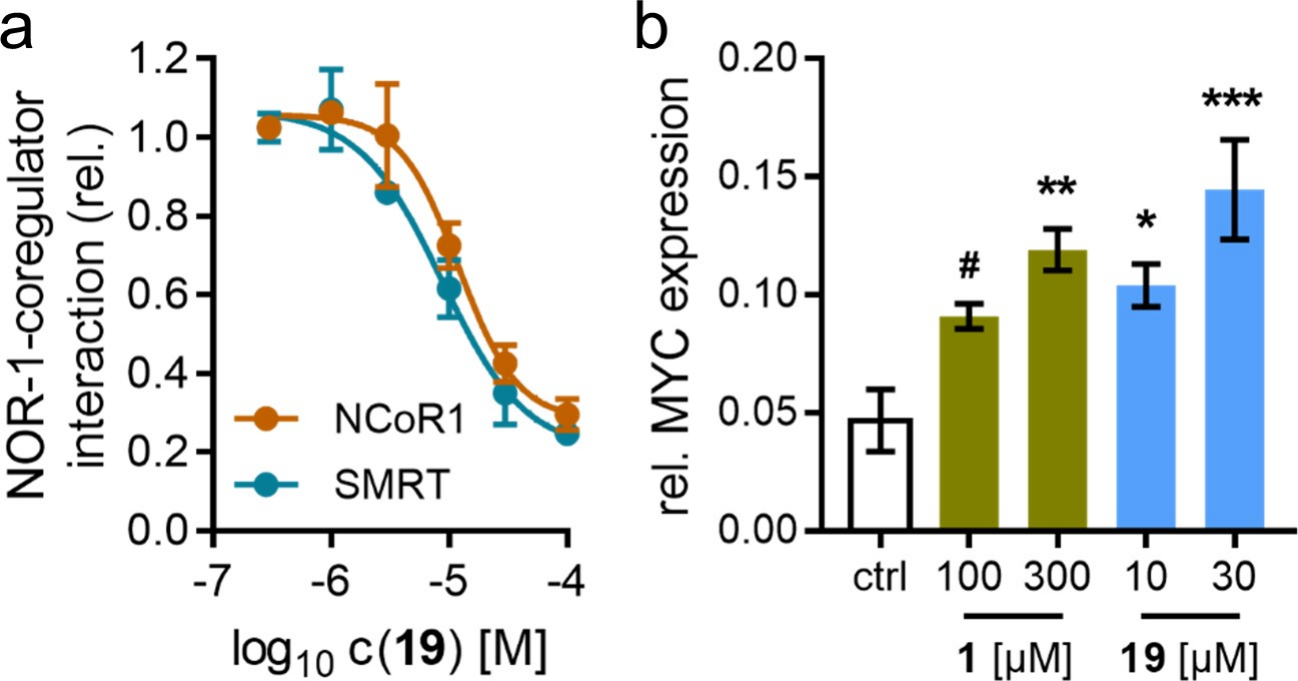
NOR‐1 modulation by the inverse agonists **1** and **19**. (a) **19** antagonized the interaction of NOR‐1 with NCoR1 (IC_50_ 12±3 μM) and SMRT (IC_50_ 9±2 μM). Data are the mean ±SEM, n=3. (b) The inverse NOR‐1 agonists **1** and **19** induced expression of the NOR‐1 suppressed c‐MYC proto‐oncogene (MYC) in HeLa cells thus confirming cellular target engagement by **1** and **19**. Data are the mean ±SEM relative MYC mRNA levels (2^−ΔCt^) with GAPDH as reference gene, n=4. ^#^ p<0.1, *p<0.05, **p<0.01, ***p<0.001 (ANOVA).

With the lack of NOR‐1 modulators to study the mechanisms and role of the transcription factor,^[24]^ the inverse NOR‐1 agonist **19** evolves as a useful early chemical tool. Therefore, we employed **19** to study mechanism of NOR‐1 modulation and cellular effects of NOR‐1 blockade (Figure 4). We have previously observed that modulation of the closely related nuclear receptor NR4A2 involves ligand‐dependent interactions with the nuclear receptor co‐repressor 1 (NCoR1) and the silencing mediator for retinoid and thyroid hormone receptors (SMRT)[^25^, ^26^] suggesting potential relevance in NOR‐1 modulation, as well. Indeed, **19** efficiently blocked the interaction of NOR‐1 with NCoR1 and SMRT (Figure 4a) with low micromolar potencies (NCoR1: IC_50_ 12±3 μM; SMRT: IC_50_ 9±2 μM).

As NOR‐1 acts as a transcription factor, we then probed the ability of the tool **19** to modulate NOR‐1 regulated gene expression. It has been shown that constitutive NOR‐1 activity directly suppresses expression of the c‐MYC proto‐oncogene (MYC).[^9^, ^10^, ^27^] In line with this observation, the inverse NOR‐1 agonist lead **1** and the more potent analogue **19** induced MYC expression in HeLa cells (Figure 4b). These results thus support target engagement of **1** and **19** in cellular setting and further highlight the suitability of **19** as early inverse NOR‐1 agonist tool.

## Conclusion

The NR4A family of nuclear receptors is gaining attention for therapeutic potential especially in neurodegeneration^[24]^ and cancer.^[9]^ While there has been considerable progress in ligand discovery for Nur77 (NR4A1) and Nurr1 (NR4A2), NOR‐1 (NR4A3) modulators are still lacking but needed as chemical tools to enable further research on the receptor′s therapeutic potential. Our results confirm that NOR‐1 activity can be modulated by small molecule ligands and provide a set of lead structures for NOR‐1 modulator development. Despite not being exhaustive, our preliminary SAR elucidation resulted in the inverse NOR‐1 agonist **19** which may be useful as an early tool compound. Aligning with its inverse NOR‐1 agonist activity and the finding that NOR‐1 directly suppresses MYC expression,^[9]^ **19** enhanced MYC expression confirming cellular target engagement and suitability for in vitro studies.

## Experimental Section

**Compounds**. The screening library and compound **1**–**10** were obtained from commercial vendors. Preparation and analytical data of **11**–**20** have been reported previously.^[28]^

**Hybrid reporter gene assays**. The reporter gene assay was performed as reported previously^[25]^ in HEK293T cells in 96‐well format using pFR‐Luc (Stratagene, La Jolla, CA, USA; reporter), pRL‐SV40 (Promega, Madison, WI, USA; control) and the Gal4‐NOR‐1 fusion receptor plasmid pFA‐CMV‐hNOR‐1‐LBD coding for the hinge region and LBD of the canonical isoform of NOR‐1 or pECE‐SV40‐Gal4‐VP16^[15]^ (Addgene, entry 71728, Watertown, MA, USA; for the Gal4‐VP16 control assay). In the primary screening, the core set of the Prestwick Drug Fragments Library was tested at 100 μM in two biologically independent repeats. All other samples were tested in duplicates in at least three biologically independent repeats. Luminescence was measured with a Spark 10 M luminometer (Tecan Group AG, Mannedorf, Switzerland), firefly luciferase data were divided by Renilla luciferase data and multiplied by 1000 to obtain relative light units (RLU). Fold activation was obtained by dividing the mean RLU of a test compound at a respective concentration by the mean RLU of untreated control. Dose‐response curves were fitted with the equation “[Agonist]/[Inhibitor] vs. response (three parameters)” in GraphPad Prism (version 7.00, GraphPad Software, La Jolla, CA, USA).

**Reporter gene assays for NOR‐1 interactions with NCoR1 and SMRT**. The interaction of NOR‐1 with NCoR1 and SMRT was studied in reporter gene assays in HEK293T cells using pFR‐Luc, pRL‐SV40 and pFA‐CMV‐hNOR‐1‐LBD in combination with either pFTI‐CMV‐NCoR1 or pFTI‐CMV‐SMRT, which code for fusion proteins composed of the transcriptional inducer VP16 and fragments of the nuclear receptor co‐regulators NCoR1 or SMRT comprising one interaction motif.^[29]^ HEK293T cells were cultured in Dulbecco's modified Eagle's medium (DMEM), high glucose, supplemented with 10 % fetal calf serum (FCS), sodium pyruvate (1 mM), penicillin (100 U mL^−1^), and streptomycin (100 μg mL^−1^) at 37 °C and 5 % CO_2_, and seeded in 96‐well plates (3×10^4^ cells/well). After 24 h, the medium was changed to Opti‐MEM without supplements and the cells were transiently transfected with above‐described plasmids using Lipofectamine LTX reagent (Invitrogen). 5 h after transfection, the medium was changed to Opti‐MEM supplemented with penicillin (100 U mL^−1^) and streptomycin (100 μg mL^−1^) containing 0.1 % DMSO with or without the respective test compound at varying concentration. After 16 h incubation, cells were assayed for luciferase activity using the Dual‐Glo Luciferase Assay System (Promega) and luminescence was measured with a Spark 10 M luminometer (Tecan Group Ltd). Each sample was tested in duplicates in three independent experiments. Firefly luciferase data were divided by renilla luciferase data to obtain relative light units (RLU) and RLU of each test sample were normalized to the negative control (0.1 % DMSO).

**Analysis of NOR‐1 regulated MYC expression in HeLa cells**. HeLa cells (ATCC CCL‐2™) were grown in RPMI 1640 supplemented with 10 % fetal calf serum (FCS), penicillin (100 U mL^−1^) and streptomycin (100 μg mL^−1^) at 37 °C and 5 % CO_2_ and seeded at a density of 250,000 cells per well in 12‐well plates. After 24 h, medium was changed to RPMI 1640 supplemented with 0.2 % FCS, penicillin (100 U mL^−1^) and streptomycin (100 μg mL^−1^) and the cells were incubated for another 24 h before stimulation with **1** (100 and 300 μM), **19** (10 and 30 μM) each with 0.1 % DMSO or with 0.1 % DMSO alone as negative control. After 16 h of incubation, the medium was removed, the cells were washed with phosphate buffered saline (PBS) and after full aspiration of residual liquids immediately frozen at −80 °C until further procession. Total RNA was then isolated using the E.Z.N.A.® Total RNA Kit I (Omega Bio‐tek, Norcross, USA) following the manufacturer's instructions. RNA concentration and purity was assessed using a NanoDrop™ One UV/VIS spectrophotometer (Thermo Fisher Scientific, Waltham, USA) at 260/280 nm. Right before reverse transcription (RT), RNA was linearized at a concentration of 133 ng μL^−1^ at 65 °C for 10 min and then immediately incubated on ice for at least 1 min. RT was performed using 2 μg total RNA, 20 U Recombinant RNasin® Ribonuclease Inhibitor (Promega, Mannheim, Germany), 100 U SuperScript ® IV Reverse Transcriptase including 5x First Strand Buffer and 0.1 M dithiothreitol (Thermo Fisher Scientific, Waltham, USA), 3.75 ng linear acrylamide, 625 ng random hexamer primers (#11277081001, Merck, Darmstadt, Germany), and 11.25 nmol deoxynucleoside triphosphate mix (2.8 nmol each ATP, TTP, CTP, GTP; #R0186, Thermo Fisher Scientific, Waltham, USA) at a maximum volume of 22.45 μL. RT was run at 50 °C for 10 min and 80 °C for 10 min using a Thermal cycler XT^96^ (VWR International, Darmstadt, Germany). Quantitative polymerase chain reaction (qPCR) was conducted using an Applied Biosystems™ QuantStudio 1 (Waltham, USA) and a SYBR green based detection method with 0.2 μL of prepared cDNA solution, 6 pmol of forward and reverse primer, 0.8 U Taq DNA Polymerase (#M0267, New England Biolabs, Ipswich, USA), 4 ppm SYBR® Green I (#S9430, Sigma Aldrich, St. Louis, USA), 1.2 nmol deoxynucleoside triphosphate mix (as indicated above), 60 nmol MgCl_2_, 4 μg bovine serum albumin (#B14, Thermo Fisher Scientific, Waltham, USA), 20 % BioStab PCR Optimizer II (#53833, Merck, Darmstadt, Germany), and 10 % Taq buffer without detergents (#B55, Thermo Fisher Scientific, Waltham, USA) topped up at a final volume of 20 μL with ddH_2_O. Samples underwent 40 cycles of 15 s denaturation at 95 °C, 15 s of primer annealing at 60 °C, and 20 s of elongation at 68 °C. PCR product specificity was evaluated using a melting curve analysis. MYC mRNA expression was normalized to GAPDH mRNA expression per each sample using the ΔCt‐method. The following primers were used: GAPDH: fw: 5’‐AGG TCG GAG TCA ACG GAT TT‐3’, rev: 5’‐TTC CCG TTC TCA GCC TTG AC‐3’; MYC: fw: 5’‐CCT GGT GCT CCA TGA GGA GAC‐3’, rev: 5’‐CAG ACT CTG ACC TTT TGC CAG G‐3’.

**Computational methods**. Molecular features (AMW, TPSA, XlogP, Murcko scaffolds), Morgan fingerprints and Tanimoto similarity were computed using CDK and RDKit software in KNIME (v4.4.0).

## Conflict of interest

The authors declare no conflict of interest.

1

## Data Availability

The data that support the findings of this study are available from the corresponding author upon reasonable request.
